# Validity of a self-reported measure of familial history of obesity

**DOI:** 10.1186/1475-2891-7-27

**Published:** 2008-09-10

**Authors:** Ann-Marie Paradis, Louis Pérusse, Gaston Godin, Marie-Claude Vohl

**Affiliations:** 1 Department of Food Science and Nutrition, Institute of Nutraceuticals and Functional Food Laval University, Quebec, Canada, and Lipid Research Center, CHUQ-CHUL Pavilion, 2705 Laurier Blvd, TR-93, Sainte-Foy, Quebec, G1V 4G2, Canada; 2 Department of Social and Preventive Medicine, Laval University, Quebec, G1K 7P4, Canada; 3 Faculty of Nursing, Laval University, Quebec, Canada, G1K 7P4 and Canada Research Chair on Behaviour and Health, Quebec, G1K 7P4, Canada; 4 Department of Food Science and Nutrition, Institute of Nutraceuticals and Functional Food Laval University, Quebec, Canada, and Lipid Research Center, CHUQ-CHUL Pavilion, 2705 Laurier Blvd, TR-93, Sainte-Foy, Quebec, G1V 4G2, Canada

## Abstract

**Background:**

Familial history information could be useful in clinical practice. However, little is known about the accuracy of self-reported familial history, particularly self-reported familial history of obesity (FHO).

**Methods:**

Two cross-sectional studies were conducted. The aims of study 1 was to compare self-reported and objectively measured weight and height whereas the aims of study 2 were to examine the relationship between the weight and height estimations reported by the study participants and the values provided by their family members as well as the validity of a self-reported measure of FHO. Study 1 was conducted between 2004 and 2006 among 617 subjects and study 2 was conducted in 2006 among 78 participants.

**Results:**

In both studies, weight and height reported by the participants were significantly correlated with their measured values (study 1: r = 0.98 and 0.98; study 2: r = 0.99 and 0.97 respectively; p < 0.0001). Estimates of weight and height for family members provided by the study participants were strongly correlated with values reported by each family member (r = 0.96 and 0.95, respectively; p < 0.0001). Substantial agreement between the FHO reported by the participants and the one obtained by calculating the BMI of each family members was observed (kappa = 0.72; p < 0.0001). Sensitivity (90.5%), specificity (82.6%), positive (82.6%) and negative (90.5%) predictive values of FHO were very good.

**Conclusion:**

A self-reported measure of FHO is valid, suggesting that individuals are able to detect the presence or the absence of obesity in their first-degree family members.

## Introduction

Familial history is a risk factor of several chronic diseases of public health significance, including obesity. Therefore, it has been proposed that familial history information could be useful in clinical practice and construction of family pedigrees could provide important data for use in genetic studies [1-3]. However, little is known about the accuracy of self-reported familial history, particularly self-reported familial history of obesity (FHO).

At present, the literature mainly provides information on self-reported height and weight. More than two decades ago, Stunkard and Albaum [4] reported that self-reported weights were remarkably accurate across different ages and sexes. Subsequent studies confirmed that self-reported values are valid and provide reliable indicators of measured weight and height [2,5-11]. Recently, Gorber and colleagues [12] have published a review of the literature determining what empirical evidence exists regarding the agreement between objective (measured) and subjective (reported) measures in assessing height, weight and body mass index (BMI) in observational and experimental studies of adult populations. Overall, this review, including 64 studies, reported an evident trend for an overestimation of height and an underestimation of weight and BMI, in both men and women [12]. Height and weight estimations from a family member (father, mother and siblings) follow the same pattern. Indeed, Reed and Price [2] conducted a study using a cohort of 374 first-degree relatives from 94 Caucasian families, to assess the value of family informant estimates of height and weight. It was shown that respondents systematically overestimated heights (mean = 1.4 cm) and underestimated weights (mean = 4.1 kg) of their family members [2]. Although the accuracy of self-reported height and weight and the accuracy of family member estimates have been studied, there is still no available data on the validity of self-reported measure of FHO. This information could be of great importance for use in genetics studies and other studies such as those designed to understand differences between subjects with and without FHO. Indeed, these studies should benefit from information about the accuracy of a self-reported measure of FHO which has the advantages of practicality and being a low cost method applicable to a large number of individuals. Thus, the aim of this study was to examine the validity of a self-reported measure of FHO. Before doing so, we first compared self-reported and measured weight and height in a cohort of 245 men and 372 women from the greater Quebec City area (study 1). Secondly, we assessed the correlation between weight and height estimations reported by a participant and the values provided by each family member (mother, father and siblings) in a cohort of 78 subjects including 199 family members (study 2). Finally, we examined the validity of a self-reported measure of FHO in the same cohort (study 2).

## Methods

### Study 1

#### Study population and data collection

Participants were adults aged between 18 to 55 years. Subjects were recruited in the Quebec City metropolitan area through public advertisements in local newspapers and by electronic messages sent to university and hospital employees. A trained research assistant conducted a telephone interview with people who responded to the advertisement messages. The assistant asked the participants to report their body weight and height. Subjects had to answer to the following questions: *What is your current weight?, What is your current height? *Following the interview, eligible participants were given an appointment within the next 2–3 weeks to come to the laboratory to meet trained research assistants for anthropometric measurements. The beam Scale with height rod graduated in centimetres was used (Detecto, Webb City, USA) to obtain a measure of weight and height of each participant. Weight was measured to the nearest 0.1 kg and height was measured to the nearest 0.5 cm. The scale was calibrated before each examination. BMI was computed as weight in kilograms divided by height in meters squared. Enrolment of the subjects took place between 2004 and 2006. The final study sample consisted of 245 men and 372 women. All subjects gave their written consent to participate into this study which has been approved by the Ethics Committee of the local university Hospital Research Center.

#### Statistical analysis

Paired Student's *t*-test was used to compare the means of self-reported and measured height and weight. Pearson's correlation coefficients were used to examine the association between the self-reported and measured height and weight. All statistical analyses were performed using SAS statistical software, version 8.2 (SAS Institute Inc, Cary, NC) and statistical significance was defined as p < 0.05.

### Study 2

#### Study population and data collection

Participants were adults aged between 18 to 55 years. Subjects were recruited as described in study 1. Enrolment of the subjects took place in 2006. The sample included 78 respondents (52 women and 26 men) and their family members (mother, father, and siblings) (n = 199). Each of the 78 volunteers was given an appointment to come to the laboratory within the next 5–6 days after the initial contact. During their visit, they were first asked to report on a self-administrated questionnaire their own weight and height and to estimate weight and height of each of their family members (mother, father and siblings). Subjects had to answer the following questions: *What is your current weight?, What is your current height?, What is the current weight of your mother, father, and siblings?*, and *What is the current height of your mother, father, and siblings?*. Second, volunteers had to identify whether any of their family members (mother, father and siblings) were obese. If the participant identified at least one obese first-degree relative, a subjective FHO was determined as positive. The subjective FHO was considered negative if no obese first-degree relative was identified. Third, the beam Scale with height rod graduated in centimetres was used (Detecto, Webb City, USA) to obtain a measure of weight and height of each participant. Weight was measured to the nearest 0.1 kg and height was measured to the nearest 0.5 cm. The scale was calibrated before each examination. BMI was computed as weight in kilograms divided by height in meters squared. Each participant was asked to transmit (if needed by mail) an informed consent and a self-administrated questionnaire to each of his/her family members, and to not discuss the measurements or the study with their family members. These family members were asked to complete the informed consent, to report their own weight and height, and to send this information back to the research team. All family members (mother, father, and siblings) were asked the following questions: *What is your current weight?*, and *What is your current height?*. This allowed the calculation of BMI for each family member (mother, father, and siblings) and to define an objective measure of FHO. Thus, objective FHO+ was defined as having at least one obese (BMI ≥ 30 kg/m^2^) family member and objective FHO- as having no family member with a BMI ≥ 30 kg/m^2^. All subjects gave their written consent to participate into this study which was approved by the Ethics Committee of the local university Hospital Research Center. Complete informations about FHO were obtained for 44 families including 199 family members.

### Statistical analysis

The student *t*-test was used 1) to compare the means of self-reported and measured height and weight and 2) to compare the means of weight and height estimations reported by the participants to values reported by each family member. Pearson's correlation coefficients were calculated to assess the linear associations between self-reported and measured height and weight. Pearson's correlation coefficients were also used to examine the relation between height and weight estimations reported by the participant and the values provided by each family member. Sensitivity and specificity of FHO were calculated. The sensitivity represents the capacity of the participant to correctly report positive FHO (correctly reported positive FHO/all subjective positive FHO) and the specificity represents the capacity of the participant to correctly report negative FHO (correctly reported negative FHO/all subjective negative FHO). Positive predictive value estimating the proportions of participants who correctly reported positive FHO (correctly reported positive FHO/all objective positive FHO) was also computed together with the negative predictive value which estimates the proportions of participants who correctly reported FHO (correctly reported negative FHO/all objective negative FHO). The likelihood ratio was calculated to estimate the ratio of the true positive rate versus the false positive rate. The ROC-Curve analysis was computed to test the capacity of the self-reported FHO to classify correctly participants. Finally, kappa coefficients were calculated to measure congruence between the subjective FHO and the objective FHO. The following classification suggested by Landis and Koch [13] was used: poor-to-fair (kappa < 0.40), moderate (kappa of 0.41 to 0.60), substantial (kappa of 0.61 to 0.80) and excellent (kappa of 0.81 to 1.00). All statistical analyses were performed in SAS statistical software, version 8.2 (SAS Institute Inc, Cary, NC) and statistical significance was defined as p < 0.05.

## Results

### Study 1

The mean (± SD) age of the 617 participants was 37.9 ± 11.3 years. Mean values of self-reported and measured values of weight, height and BMI are presented in Table 1. In men, self-reported and measured values were not significantly different (p > 0.05). In women, the mean difference between self-reported and measured weight and BMI were not significantly different. However, women overestimated their height by a mean of 1.2 cm (p < 0.05). Differences observed between self-reported and measured values were similar in men and women (p > 0.05). Strong correlations between self-reported and measured weight, height and BMI were observed (r = 0.99, p < 0.0001; 0.97, p < 0.0001; r = 0.97, p < 0.0001, respectively) (data not shown).

**Table 1:** Mean (± SD) self-reported and measured anthropometric measurements and their differences in men and women of the study 1 and study 2.

	Self-reported	Measured	Difference
**Study 1**			
Men (N = 245)			
Weight (kg)	86.85 ± 17.73	87.00 ± 18.86	0.15 ± 3.27
Height (cm)	176.77 ± 6.72	175.60 ± 6.90	-1.17 ± 2.3
BMI (kg/m^2^)	27.73 ± 5.02	28.17 ± 5.54	0.43 ± 1.37
Women (N = 372)			
Weight (kg)	69.98 ± 14.99	70.61 ± 16.62	0.62 ± 2.79
Height (cm)	163.38 ± 6.59	162.18 ± 6.46	-1.20 ± 2.21*
BMI (kg/m^2^)	26.23 ± 5.43	26.88 ± 5.87	0.65 ± 1.36
**Study 2**			
Men (N = 26)			
Weight (kg)	80.35 ± 10.83	80.86 ± 11.36	0.51 ± 1.28
Height (cm)	175.17 ± 6.84	176.88 ± 6.72	1.71 ± 1.91
BMI (kg/m^2^)	26.21 ± 3.39	25.88 ± 3.63	-0.32 ± 0.57
Women (N = 52)			
Weight (kg)	64.96 ± 14.34	65.93 ± 16.17	0.96 ± 4.44
Height (cm)	161.42 ± 6.51	163.12 ± 6.78	1.70 ± 1.69
BMI (kg/m^2^)	24.97 ± 5.53	24.86 ± 6.42	-0.11 ± 2.17

*Significantly different from the self-reported value, p < 0.05.

### Study 2

The mean (± SD) age of the 78 participants was 31.7 ± 10.4 years whereas the mean age of family members was 45.0 ± 15.7 years. On average, each family had 4.5 ± 1.1 members with a minimum of three and a maximum of eight family members. Participants of study 2 were comparable to those of study 1. Despite that participants of study 2 were slightly younger, they had similar weight and BMI (after adjustment for age and sex, data not shown). The mean values of self-reported and measured weight, height, BMI as well as their differences are presented in Table 1. No significant differences were observed between self-reported and measured weight and BMI. Correlations between self-reported and measured weight, height and BMI were observed (r = 0.98, p < 0.0001; 0.98, p < 0.0001; r = 0.95, p < 0.0001, respectively) (data not shown).

To determine the accuracy of estimations provided by the participant about the weight, height, and BMI of their family members, values reported by the study participants were compared to weight, height, and BMI reported by each family member. Mean values of weight, height and BMI are presented in Table 2. No significant differences were observed between informant estimates and family member self-reported values. In addition, highly significant correlations were observed for weight, height, and BMI reported by the participants and self-reported values of family members (weight: r = 0.96, height: r = 0.95, BMI: r = 0.91 p < 0.0001) (data not shown).

**Table 2:** Self-reported and family informant estimates of height, weight and body mass index (BMI) (study 2).

	Self-reported values	Informant estimates	Difference
Weight (kg)	72.72 ± 16.55	71.90 ± 16.00	0.81 ± 4.87
Height (cm)	166.91 ± 9.32	166.35 ± 8.92	0.56 ± 3.01
BMI (kg/m^2^)	26.04 ± 5.23	25.92 ± 5.05	0.12 ± 2.15

For intrinsic and predictive validity, the distribution of individuals according to the objective and subjective measure of FHO are presented in Table 3. Sensitivity was 90.5%, whereas specificity was 82.6%. The positive and negative values were respectively 82.6% and 90.5% and the likelihood ratio was 5.2. The area under the ROC-curve was 0.87 indicating that the classification into FHO categories was excellent (not shown). Finally, the validity of the self-reported measure of FHO was verified using the kappa statistic. Considering the 44 participants, kappa analysis indicated that the degree of agreement between the subjective measure of FHO reported by the participants and the objective measure derived from the self-reported BMI of family members was substantial as indicated by the value of 0.72. The degree of agreement was also verified separately for men and women, age groups, and body weight categories. Kappa values of 0.68 and 0.85 were observed respectively for men and women. For age (median split), a substantial degree of agreement was observed in younger individuals (<28 years) (kappa = 0.63) and an excellent degree of agreement (kappa = 0.81) was observed in older individuals (≥ 28 years). With respect to body weight, there was a moderate degree of agreement in heavier individuals (median split, BMI ≥ 23.8 kg/m^2^, kappa = 0.58) and a substantial degree of agreement in individuals having a BMI < 23.8 kg/m^2 ^(kappa = 0.71).

**Table 3:** Distribution of individuals according to the measure of the subjective and objective FHO.

	Objective measure of FHO		
		
	Positive	Negative	Total
Subjective measure of FHO			
Positive	19	4	23
Negative	2	19	21
Total	21	23	44

## Discussion

Although familial history information is collected in clinical and research studies, little information is available on the validity of a proband's reported familial history. To our knowledge, this is the first study in the field of obesity which investigates the validity of a self-reported measure of FHO. Before doing so, we first verified that individuals were able to adequately report their own weight and height. Thus, the accuracy of self-reported weight and height has been first examined in study 1. Results obtained are concordant with the literature which has demonstrated that self-reported height and weight are highly predictive of measured values [2,5-9]. In contrast to some previous studies [2,5,8,9,14,15] and a recent review [12], similar means of self-reported and measured weight, height (except for women), and BMI were observed in men and women of study 1. In women, self-reported values of height were overestimated. The overestimation was minor (1.2 cm) and did not significantly affect the mean BMI values (self-reported versus measured values). Similar analyses were performed in a second study sample (study 2) and no significant differences were observed between self-reported and measured values in both men and women.

Regarding weight and height estimations of family members provided by the participants in study 2, high correlations were observed between estimations reported by the participants and the values reported by each family member. Moreover, no significant difference was observed between the values reported by the participants and the one reported by each family member. The use of self-reported current weight and height for each family member instead of the use of measured values could be considered as a limitation of the present study. However, no major difference in self-reported and measured weight, height with (except among the women of study 1) and BMI were reported in participants from two different samples. Thus, we assume that family members had also truthfully reported their weight and height.

Although results of the present study suggest that individuals were able to adequately report their own weight and height and those of their family members, it is important to mention that the major aim of the present study was to examine the validity of a self-reported measure of FHO. By comparing, subjective FHO (self-reported by the participant) with objective FHO (defined by the self-reported BMI of family members), we found that objective FHO are close to perfectly reliable. Very good sensitivity values were observed indicating that individuals with positive FHO correctly reported the presence of FHO. The high level of specificity observed indicates that individuals without FHO correctly reported the absence of FHO. The positive and negative predictive values were high, suggesting that the method use in this study to determine the presence or the absence of FHO is reliable. In other words, individuals are able to report the presence or the absence of obesity in their first-degree family members. It is important to note that substantial or excellent agreement was also observed when kappa statistic was calculated according to gender and age. Moreover, substantial and moderate degree of agreement was observed depending of weight status.

The present study has several limitations. First, since demographic characteristics can have an influence on the degree of reporting error [12] and that bias of estimated heights and weights are influenced by informant characteristics [2], it would be relevant to perform statistical analyses after stratification on the basis of gender, age, education, weight, etc. However, the sample size of the study 2 is relatively small for such a stratification. Other studies with a larger number of families are needed to examine this point. Second, the determination of the objective measure of FHO was available only for families in which all members have completed the questionnaire. It is possible that these families may be more willing than non-respondent families to provide precise weight and height. Third, most of the participants may have been aware that stature and weight would be measured following the self-reported, which could have mitigated misreporting and could minimise errors between self-reported and measured values which usually tend to deviate towards a 'preferred' body size [16]. This bias could explain why self-reported and measured weight, height (except for women in study 1), and BMI were similar in the present study in contrast to other studies [2,5,8,9,12,14,15].

In conclusion, although the results of the present study need to be replicated in other cohorts or populations with larger number of families, the present study reports important findings. Subjects can accurately self-report their weight and height and those of their family members. More importantly, the results of this study indicate that a self-reported measure of FHO is valid, suggesting that individuals are able to detect the presence or the absence of obesity in their first-degree family members. This finding is important for future research. Indeed, since the presence of a positive FHO has been highlighted as a predictive risk factor for the development of weight excess [17-22], and because genetic counselling is done in clinical settings, obtaining an accurate FHO is essential. Studies such as those designed to understand difference between subjects with and without FHO should benefit from information about the accuracy of self-reported FHO.

## Competing interests

The authors declare that they have no competing interests.

## Authors' contributions

AMP performed the statistical analysis of the data and took the primary role in drafting the manuscript. LP, GG and MCV guided the strategy of the data analysis, assisted with the interpretation of the results, and provided critical review of the manuscript. LP, GG and MCV conceived the study. All authors read and approved the final manuscript.
